# Catch per Unit Effort, Density and Size Distribution of the Oysters *Pinctada capensis* and *Saccostrea cucullata* (Class Bivalvea) on Inhaca Island, Southern Mozambique

**DOI:** 10.3390/life13010083

**Published:** 2022-12-28

**Authors:** Mizeque Julio Mafambissa, Celia Angelica Gimo, Carlos Pestana Andrade, Adriano Afonso Macia

**Affiliations:** 1 Department of Biological Sciences, Faculty of Sciences, Eduardo Mondlane University, Maputo 1100, Mozambique; 2 Mariculture Centre of Calheta, 9370-133 Calheta, Madeira, Portugal

**Keywords:** intertidal resources, pearl oyster, rock oyster, coastal communities, Western Indian Ocean

## Abstract

Oysters are important resources for the daily diet, a source of economic income for many coastal communities and a delicacy for the tourism industry. In this study, the oysters (*Pinctada capensis* and *Saccostrea cucullata*) were investigated with the aim to assess the catch per unit effort, density and size composition. The study was conducted over a three-year period on Inhaca Island, Southern Mozambique. For both species, perception of fishery trends from collectors was carried out through habitat censuses and interviews. Transects, quadrats and daily fisheries catches approaches were used. Results show that *P. capensis* is the most exploited on the island. A total of 72.1% of respondents pointed that the oyster *P. capensis* is decreasing, due to excessive catching (75.4%) followed by natural death (24.6%), while 20.9% affirmed that the resource is stable and 7.0% are unaware about the resource trend. Oyster densities, sizes and catches per unit effort were higher in less accessible areas only for *P. capensis*. The present study provides valuable baseline information to recommend best practices to improve the exploitation, and access the need for introduction of aquaculture, towards the sustainable management and conservation of oysters, and ultimately to ameliorate people’s livelihoods.

## 1. Introduction

Oysters are widely distributed around the world. Some oysters are key species for the ecology of coastal and estuarine areas [1]. They provide important ecological services such as improving water quality, helping stabilize the coastline and protecting erosion by building their reefs, and they are also an important product of fisheries and aquaculture in many countries [2].

In tropical regions, oysters occur in rivers and coastal areas, with most species assembled in narrow bands or dense banks at a tidal range where desiccation, fouling and predation are minimized [3]. Oysters are targeted by small-scale fisheries and their harvest is usually handmade by residents, mainly by women and children, at the spring low tide period [4,5].

Oysters contribute as a source of animal proteins and to the income of coastal communities. Not surprisingly, their increasing demand in recent years has promoted a global decline on stocks due to overfishing [6], pollution, habitat destruction and disease, especially in Africa where small-scale fishing is considerably unregulated [1,7].

Mozambique has a long coast with a wide diversity of habitats, providing a variety of resources, which include fisheries’ resources, supporting the livelihood of more than half of the coastal population [8]. Among the fisheries’ resources, the most collected bivalves are clams and oysters. The importance of each group varies depending on their abundance and the needs in different areas [9]. In southern Mozambique, the sand oyster or pearl oyster *Pinctada capensis* is found associated to seagrass beds, and the rock oyster *Saccostrea cucullata* is associated with rocky shores [8,10], and considered the most harvested bivalves with economical relevance [11].

On Inhaca Island, situated 32 km from Maputo city, the exploitation of *P. capensis* and *S. cucullata* in intertidal areas has been carried out for several generations and probably for some centuries [12]. Oyster harvesting is one of the main subsistence activities practiced by the population living on this small island. There is a great dependence of the population on the resource due to the lack of subsistence alternatives [13].

Besides this provisioning role to the population, oysters and oyster reefs usually provide other relevant ecosystem services, namely, water quality improvement, seashore stabilization, carbon burial, habitat provisioning for mobile fish and invertebrates, habitats for epibenthic fauna and diversification of the landscape [14]. Human exploitation can affect the distribution and abundance of bivalve populations, which can compromise the ecosystem and fisheries’ management approaches [15].

Despite their biological and socioeconomic importance on Inhaca Island, oysters have not been studied to assess their abundance, size distribution and perceptions of the status of stocks by fishermen. Therefore, the present study investigated trends in *P. capensis* and *S. cuculata* abundance, size distribution and catch-per-unit effort over a 3-year period (January 2015–December 2016 and January–December 2019). This information is critical to improve knowledge of the fisheries and support management decisions regarding the sustainable exploration of these living resources.

## 2. Materials and Methods

### 2.1. Study Area

**Figure 1:**
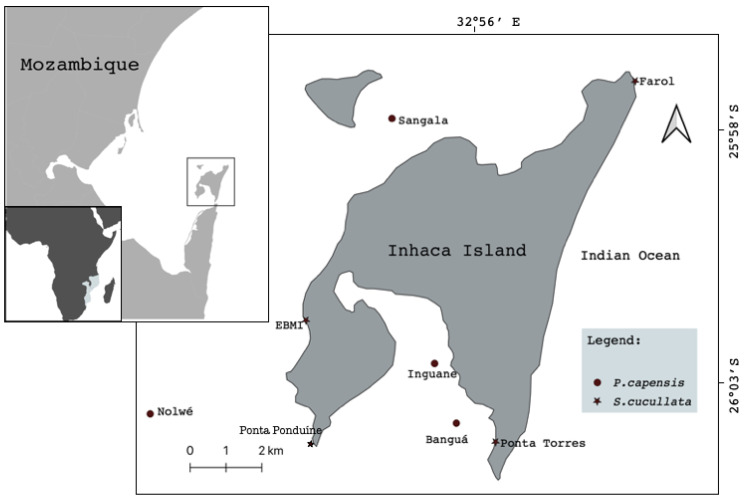
Map of study area showing sampling sites.

The study was carried out on Inhaca Island (latitude 26°07′ S, longitude 32°56′ E), southern Mozambique, located 32 km off Maputo City, Mozambique (Figure 1). The Island has a total area of approximately 42 km^2^ and is part of the Ponta de Ouro Partial Marine Reserve. It is located on the border between the shallow Bay of Maputo and the open waters of the Indian Ocean, in a transition zone from tropical to sub-tropical climate, which creates a rich diversity of both terrestrial and marine ecosystems [16].

There are two distinct seasons: the hot and rainy season (from November to April) and the cold and dry season (from May to October) with an average air temperature of 23 °C, and the sea water temperature varying from 18 °C to 22 °C and 23 °C to 32 °C for the cold and hot season, respectively [12]. The eastern part of the Island is characterized by strong currents and waves while the western part is more protected [17]. The tides are semidiurnal and have maximum amplitudes of about 3.1 m in high spring tides [16]. During low tide, a large stretch of beach is exposed, making it an interesting site for the collection of many invertebrates, including oysters [10,17].

The sampling sites were defined along the eastern and western sides of the Island and comprised eight main study areas: 4 in the seagrass meadows (*P. capensis*) and 4 in rocky shore (*S. ucullate*), (Figure 1). Two sites, selected (Sangala & Inguane) for *P. capensis* sampling, were located along the intertidal accessible during low tides, while Nolwe and Banga are located in a bank reachable only by boat during low tides. For *S. cucullate,* both sites were on rocks along the intertidal areas with direct accessibility.

### 2.2. Data Collection

Data on oyster exploitation were collected using two different approaches in a sub-tropical setting on Inhaca Island. Semi-structured interviews with the local oyster harvesters were conducted using questionnaires to gather data on the socio-economic value of these species. Overall, 73 harvesters were interviewed in the course of the study: 63 harvesters regarding *P. capensis* while only 10 harvesters for *S. cucullata*. The lower number of harvesters for *S. cucullata* is representative with regard to the frequency in the area and reflects the low interest to exploit this species due to the difficult collecting method and being time consuming. Questions focused on obtaining data on harvesting effort (numbers of harvesters per site), catch per harvester and perception of the current state of the resource. Catch (kg) per harvester was obtained by weighing the total product collected daily.

Data obtained during the application of the questionnaires was used to select sites for field work. Sampling was conducted over a three-year period (January 2015–December 2016 and January–December 2019) within four seagrass banks for the pearl oyster *P. capensis*, and at four rocky shore habitats for *S. cucullala*. Samples were collected every spring tide (full and new moons) during the study period. Species abundance, composition in size as well as catch per unit effort were evaluated. In general, subsequent sampling was done at sites that were utilized for harvest. At each selected sampling site, quantitative data were collected using a systematic (0.5 × 0.5 m) quadrats-based approach, placed along randomized 10 m transects (parallel to the coastline as well from the artisanal daily catches). The rocky shore species *S. cucullata* was sampled in the selected locations of the Island by means of (0.5 × 0.5 m) quadrats using a digital camera and samples from the artisanal daily catches. Samples from the digital camera were processed using the Image J Program software version 1.43. Overall, 5 random transects were made for each pearl oyster occurrence site and 5 transects on each site of occurrence of rocky shore oysters.

In each sampling site for both species, biomass per collector (Kg/person/day), number of collectors and oyster sizes were recorded from the catches of the artisanal collectors.

The abundance for each oyster species was assessed determining the density (ind/m^2^) by counting all individuals present in the quadrats allocated along the transect.

Shell size (dorsoventral measurement or shell length for *P. capensis* and dorsoventral measurement or shell height for *S. cucullata*) was measured with a caliper to 0.01 mm precision. Shell sizes were measured for all living individuals present in the quadrats and 30 individuals selected randomly from the catches of each collector interviewed. The mean shell size and size distribution frequency were compared between the sampling sites.

### 2.3. Data Analysis

For statistical analysis, all monthly data recorded in this study was pooled by sampling site over the 3 years sampling period.

Percentages and frequencies were used to analyze the harvester’s perception level of the current state of the resources. For quantitative data prior to the analysis, variables (density, length and CPUE) were checked for homogeneity of variances using Cochran’s C test and data were transformed as required. One way ANOVA was used to compare variations in density, length and CPUE between sites for *P. capensis* followed by Turkey’s post hoc test [18] to assess the significance of difference in the variables (density, length and CPUE). The Kruskall-Wallis non-parametric test (H) was used to compare the *S. cucullata* densities, as data were not normally distributed after transformation [18]. The values of the mean shell size were also compared between the sampling sites. To compare the mean shell size among the sampling sites for *S. cucullata*, one-way ANOVA was used followed by Turkey’s post hoc test. T-student tests were used to compare *P. capensis* mean shell size obtained from sampling and oyster harvesters. CPUE (Kg/Harvester/day) per site was estimated after the sum of all weighted catches divided by the number of harvesters. The results are represented as a mean (±standard deviation) and the significance level used for the tests was *p* = 0.05. Statistical analysis was performed using SPSS for windows version 20.

## 3. Results

### 3.1. Interviews

The results obtained in this study indicate that the most exploited oyster species on Inhaca Island is the seagrass pearl oyster *P. capensis*. According to the questionnaire, the exploitation of oysters on Inhaca Island is dominated by women (more than 90%). Age of collectors varied from 15 to 58 years old with an average age attaining 37 years. The activities of oyster harvesting and other invertebrates is mostly done during spring low tides.

**Figure 2:**
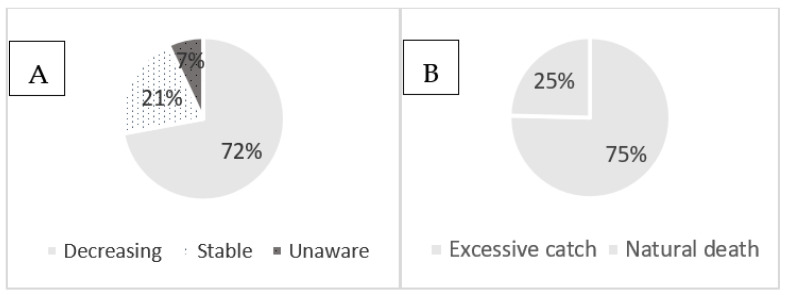
Perception of oyster harvesters about the trend (**A**) and cause of decreasing (**B**) of the resources on Inhaca Island.

Of the 73 respondents in the interviews, about 72.1% answered that the pearl oyster *P. capensis* is decreasing in number due to excessive catching (75.4%), followed by natural death (24.6%), while 20.9% meant that the resource is stable and about 7% were unaware about the resource trend (Figure 2A,B).

### 3.2. Field Sampling

The densities recorded during the three year period of study for both species are presented in Table 1. Maximum density for pearl oyster *P. capensis* with 14 ± 6 ind/m^2^ was recorded at Inguane and lower density occurred at Sangala with 4 ± 3 ind/m^2^. The density of *S. cucullata* was higher than *P. capensis*, with higher values recorded at EBMI compared to other sites. There were statistically significant differences among sites in density for both oyster species (*p* < 0.05).

Size distribution of the oysters for both species varied among different sites. Overall, for *P.capensis* oysters, length varied from 19 at Sangala to 88.8 mm at Nolwe (Table 2). Higher mean size length was recorded at Nolwe while the lower mean size was found at Bangue (Table 2).

*S. cucullata* varied in length from 13 at EBMI to 79.4 mm at Ponta Torres (Table 3). The higher mean size for this species was recorded at Ponta Torres while the lower mean size was reported at EBMI (Table 3).

**Figure 3:**
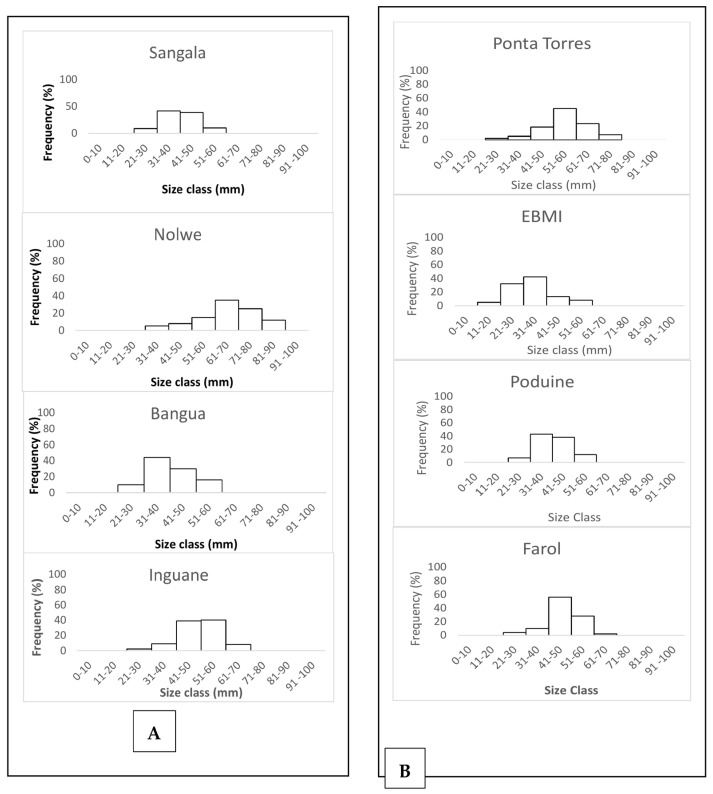
Length frequency distribution of the shells for *P. capensis* (**A**) and Height frequency distribution of the shells for the rocky shore oyster *S. cucullata* (**B**) from the sampling sites on Inhaca Island.

Oysters of smaller sizes were recorded in the shallowest sites such as the seagrass banks of Bangua and Sangala (Figure 3). For *S. cucullate*, individuals larger than 70 mm were recorded at Ponta Torres. Other sites such as Farol and EBMI recorded the highest number of oysters smaller than 40 mm.

Oyster shell size frequencies varied from 21–30 mm size intervals to 81–90 mm for *P. capensis* (Figure 3A) while for *S. cucullate*, the size frequencies varied from 11–20 mm to 71–80 mm along the sampling sites (Figure 3B).

The modal size frequencies varied between sites. Nolwe presented the higher modal size at 61–70 mm, for Bangue and Sangala at 31–40 mm while Inguane was at a 51–60 mm size interval for *P. capensis* (Figure 3A). For *S. cucullata* oysters, higher modal size frequency was recorded at 51–60 mm at Ponta Torres, 31–40 mm at EBMI and Ponta Punduine, while for Farol, they were at a size interval of 41–50 mm (Figure 3B).

**Figure 4:**
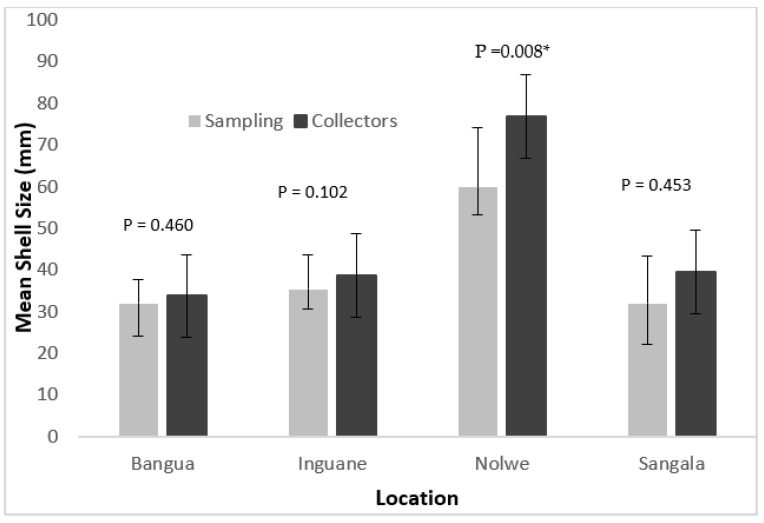
Mean shell size of the oyster *P. capensis* recorded from quadrats sampling and from the harvesters in the sampling sites on Inhaca Island. (* There was statistical significance difference).

Overall, mean shell sizes of oysters harvested by collectors were higher than the oysters collected from the sampled quadrats. However, statistical analysis only showed significant differences between the sizes of *P. capensis* collected at the Nolwe site (*p* < 0.05) (Figure 4).

In the shallower areas (Bangua, Inguane and Sangala), there were no significant differences between the oyster size harvested by collectors and that recorded in the field (*p* > 0.05) (Figure 4).

**Figure 5:**
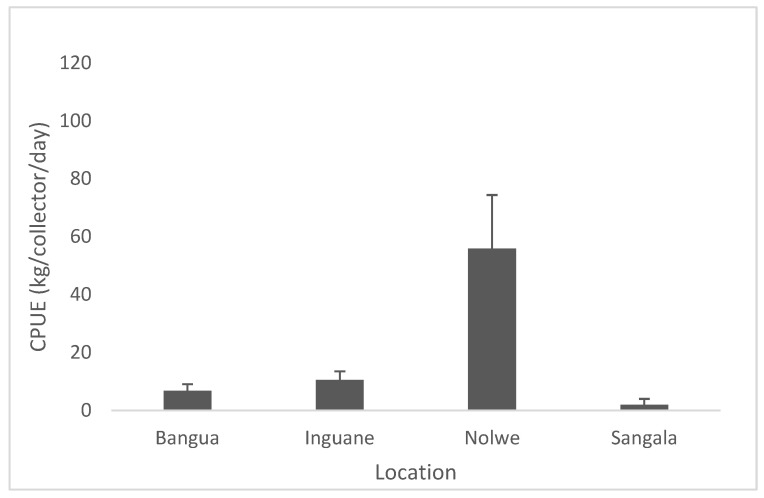
Capture per unit effort of *P. capensis* from the sampling sites recorded on Inhaca Island.

Capture per unit effort of *P. capensis* from the sampling sites on Inhaca Island are presented in Figure 5. The CPUE along the sites showed very low catches in 3 sites (Sangala, Inguane and Bangue 7–15 Kg/collector/day) comparatively to Nolwe where catches attained a 5–10 times higher number (75 kg/collector/day), Figure 4. Catch per unit effort (CPUE) among sites was significantly different (Anova I, *p* < 0.05).

As there was a low preference for collecting the rocky shore oyster *S. cucullata*, it was not possible to determine catch per unit effort for each sampling sites for this species.

## 4. Discussion

This study is the first attempt to assess catch per unit effort, density and size composition of the pearl oyster *P. capensis* and rocky shore oyster *S. cucullata* occurring at the coastal areas of Inhaca Island, Mozambique.

The densities recorded in this study for *P. capensis* (4–14 oyster/m^2^) were lower than those recorded in other regions of Mozambique such as Vilanculos and Inhassoro with 9–20 oyster/m^2^ [19], Bazaruto Island with 21–260 oyster/m^2^ [20] and in other geographic regions such as the Qatar Gulf of Arabia with 32–45 oyster/m^2^ [21], EL Gimsha bay of Red Sea with 164 oysters/m^2^ [22] and Venezuela with 16 to 50 oyster/m^2^ [23]. Two explanations could be given for this. Firstly, intensive searching for this species in intertidal areas during spring tides, as reported previously for the area [12], increases the total stress load of the substrates and thereby affects the abundance. Secondly, both *P. capensis* and *S. cucullata* have specific environmental and physical habitat requirements such as the type of sediment, habitat composition and hydrodynamic condition of the site. These factors are crucial for the abundance and distribution of benthic invertebrates [24] and may not be optimal at the studied sites. Other factors that can influence the density of oysters are predation in their natural environment as well as mortality, larval dispersion [25] and lack of appropriate substrate for settlement [26].

The higher densities recorded in Nolwe and Inguane are explained by low harvesting pressure, due to their remote location and difficult access. The remaining areas are located near the village, where they are closer and more accessible by the communities living on Inhaca island. The decrease in abundance of the pearl oyster *P. capensis* throughout the study period was evident, even in fishing grounds, which in the past yielded high densities of oysters [12]. This suggests that the level of harvesting observed during the present study have negative implications for the oysters’ stocks.

In contrast, there was no evidence of decreasing abundance and size of individuals of the rocky shore oyster *S. cucullata* during the study period. This is probably due the lower level of exploitation of this species on Inhaca.

Previous studies suggest that decrease in oyster mean shell size is also an indicator of stock decline and overfishing [12]. The average size of oysters recorded on Inhaca in this study for *P. capensis* (39.75 ± 5 mm) was lower than those recorded in the same area in 2000 where the size was 48.8 ± 4.3 mm [13] and from other regions of the country such as Vilanculos and Inhassoro, 55 ± 7.1 mm [20]; Bazaruto Island, 47.3 ± 3 mm [27]. Oysters over 40 ± 6.2 mm in size were lower in number in all sampled sites except for Nolwe bank. The low number of large size pearl oysters observed during this study is consistent with the results of the survey undertaken on Salomon Island, Australia [28]. On the other hand, there was a tendency for reduction in the percentage of oysters of larger size (>40 mm) during the study period, which resulted in the reduction of the average size in all sampled sites. The cause of this reduction may indicate that the stock is being intensively exploited. The decrease of mean size of oysters from difficult access sites to ease access sites suggests a negative impact of unregulated exploitation of oyster stocks. This finding is supported by evidence from South Africa where size of oyster specimens of protected zones was higher than oysters of unregulated zones in relation to human pressure degree [29]. A similar trend is reported for mussels whose size was smaller in open access zones than in protected sites [30]. Despite Inhaca Island being a marine protected area, the harvesting of oysters is open to the communities without restrictions for capture.

Data obtained from the questionnaire of the collectors suggests that the pearl oyster *P. capensis* is decreasing in the natural banks when compared to its yield in the past. According to [11,25], human exploitation is a factor that can disturb the distribution and affect the reproduction, growth and development of the bivalves. Pearl oysters of larger mean shell size were more abundant in the catch from the harvesters than those obtained from field sampling. This suggests selectivity of oyster harvesters preferring larger oysters and corroborates previous observations [12]. Despite lesser oysters ever reaching adult size, they reach the maturity stage very early, starting at 6 months (about 27 mm) and larvae production may not be affected [31,32,33]. Similarly, to other studies, there is evidence that pearl oyster population can be reduced to a point at which recovery is barely possible [28]. The yield is affected, since it is in the second year of the life cycle that pearl oysters considerably increase their body weight [34,35].

The variations of size frequency distribution among sites indicate a higher proportion of smaller oysters in shallower and accessible sites; it is hypothesized that in absence of size selection, indiscriminate harvesting is occurring. The consequent dominance of smaller individuals in shallower accessible sites constitutes a threat for the oyster population in the future [30].

Throughout the world, unregulated harvesting of bivalves is known to adversely affect wild stocks [29]. During the field-sampling period, we noted harvesting pressure on the pearl oyster *P. capensis*. Additionally, in some shallower and accessible areas, there is evidence of unselected oyster harvesting. In the absence of minimum oyster size, the exploitation by harvesters targets all sizes, especially immature oysters, affecting natural populations to self-recruit, and leading to overexploitation [36,37].

A community-based management approach of exploited oyster populations is considered as one of the most promising ways to link sustainability and economic growth [38]. Appropriate management strategies are required to overcome threats to pearls oysters on Inhaca Island with the support and involvement of the local community. Previous studies have demonstrated that size limits can be established as useful tools for harvest regulations, mainly in cases where management data was not available [37], such as in the case of Inhaca island. Maximum catch per person, closed harvesting seasons or areas are among other most-used measures for protection and sustainable management of oyster fisheries [39].

Another important management measure to implement would be to return the empty shells to the intertidal areas. It has been demonstrated that conspecific shells were preferred substrates for larval settlement and stock recovery [40].

Despite the multitude of stressors that can impact oyster populations and oyster reefs such as overharvesting, pollution and diseases [7], the anthropogenic pressure from catchers is likely the most relevant for the decline of abundance, individual size and catch per unit effort of *P. capensis* on Inhaca island. Our study involved the perception of harvesters regarding their harvesting habits and awareness about the condition of stocks. They are an important ecosystem component to consider when implementing management and monitoring measures for the sustainable exploitation of the oyster resources. The analysis presented here contributes to improved knowledge on both *P. capensis* and *S. cucullata* fisheries and for an urgent call for the introduction of harvesting management measures for the former species. Hopefully, it will provide an incentive to value the whole ecosystems’ services provided by the oyster habitats at the local level.

## Figures and Tables

**Table 1:** Mean (±SD) density of the oyster *P. capensis* and *S. cucullata* recorded in each sampling sites on Inhaca Island. (Different letters in the same column indicate significant differences *p* < 0.05).

*Pinctada capensis*		*Saccostrea cucullata*	
Site	Density (ind/m^2^)	Site	Density (ind/m^2^)
Bangua	11 ± 2.8 ^a^	EBMI	61 ± 7 ^a^
Inguane	14 ± 6 ^a^	Farol	50 ± 5 ^b^
Nolwe	11 ± 3.5 ^a^	Ponta Ponduine	35 ± 4 ^c^
Sangala	4 ± 3 ^b^	Ponta Torres	51 ± 8 ^b^

**Table 2:** Mean (±SE), minimum and maximum shell length of pearl oyster *P. capensis* in the sampling sites on Inhaca Island. (Different letters in the same column indicate significant differences *p* < 0.05).

Site	Mean ± SE (mm)	Minimum (mm)	Maximum (mm)
Bangua (n = 364)	31.9 ± 9.9 ^a^	21.5	56.6
Inguane (n = 466)	35.3 ± 8.3 ^a^	19	70.2
Nolwe (n = 360)	59.8 ± 18.6 ^b^	24.9	88.8
Sangala (n = 176)	32 ± 17.8 ^a^	20.7	60

**Table 3:** Mean (±SE), minimum and maximum shell height of rocky shore oyster *S. cucullata* in the sampling sites on Inhaca Island. (Different letters in the same column indicate significant differences *p* < 0.05).

Site	Mean ± SE (mm)	Minimum (mm)	Maximum (mm)
EBMI (n = 105)	30.1 ± 6.2 ^a^	13	51.3
Farol (n = 245)	30.6 ± 7 ^a^	21.6	63
Ponta Punduine (n = 102)	33.2 ± 6.9 ^a^	22.9	55.4
Ponta Torres (n = 106)	42.9 ± 8.8 ^b^	24	79.4

## Data Availability

Not applicable.
